# A Parent-Focused Intervention Programme to Promote Socio-Emotional Well-Being of Preschool Children: “Hug and Change”

**DOI:** 10.3390/healthcare14183014

**Published:** 2026-09-15

**Authors:** Athina Vatou, Anastasia Vatou, Maria Evangelou-Tsitiridou, Eleni Tympa, Athanasios Gregoriadis

**Affiliations:** 1 Department of Early Childhood Education and Care, International Hellenic University, 570 01 Thessaloniki, Greece; 2 School of Early Childhood Education and Care, Aristotle University of Thessaloniki, 541 24 Thessaloniki, Greece; asis@nured.auth.gr

**Keywords:** socio-emotional well-being in early childhood, parent-focused intervention, psychosocial adjustment, social competence, parent–child relationship

## Abstract

**Highlights:**

**What are the main findings?**

- The *Hug and Change* programme improved children’s psychosocial development and social competence in early childhood.
- Parent-focused interventions can effectively promote children’s socio-emotional well-being during early childhood.

**What are the implications of the main findings?**

- Supporting parents through structured, evidence-based interventions may enhance children’s socio-emotional well-being by strengthening everyday parent–child interactions.
- Integrating evidence-informed parent-focused programmes into preschool settings may expand access to preventive support while strengthening family–school partnerships.

**Abstract:**

**Background/Objectives**: Socio-emotional well-being during early childhood is closely associated with children’s later psychosocial adjustment, social competence, and overall developmental outcomes. Parents play a central role in fostering these competencies through everyday interactions, highlighting the importance of parent-focused interventions. This study aimed to evaluate the effectiveness of *Hug and Change*, a theory-informed parenting intervention developed for the Greek context, in promoting preschool children’s socio-emotional well-being by improving psychosocial adjustment and social competence. **Methods**: A quasi-experimental pretest–posttest design with an intervention and a comparison group was employed. The study included 123 parents of preschool children aged 4–5 years attending public preschools in Greece. Parents in the intervention group participated in the *Hug and Change* programme, which consisted of ten weekly group sessions combining parent-focused learning with structured parent–child activities designed to strengthen everyday parent–child interactions. Children’s psychosocial adjustment was assessed using the parent version of the Strengths and Difficulties Questionnaire (SDQ) while social competence was measured using the parent version of the Early School Behaviour Scale (ESBS). Outcomes were assessed before and immediately after the intervention. **Results:** The experimental group showed significant improvements from pre- to post-intervention on the SDQ and Social Competence, with reduced behavioural difficulties and increased prosocial behaviour and social skills; no significant changes occurred in the comparison group. Between-group comparisons confirmed significantly greater gains in the experimental group for the study’s variables. **Conclusions**: The findings suggest that the *Hug and Change* programme represents a promising parent-focused intervention for promoting preschool children’s socio-emotional well-being. Strengthening parenting practices and the quality of everyday parent–child interactions may contribute to improvements in both psychosocial adjustment and social competence, highlighting the potential of parent-focused interventions implemented within early childhood education settings.

## 1. Introduction

Early childhood represents a critical period for human development, during which the foundations of lifelong health, learning, behaviour and well-being are established. The World Health Organization emphasises that nurturing care and responsive caregiving are essential for enabling children to achieve their developmental potential and promoting health and well-being [1]. Contemporary perspectives further conceptualise child well-being as a multidimensional construct encompassing children’s psychological, social, emotional, cognitive and physical functioning rather than merely the absence of developmental difficulties [2,3]. Within this framework, psychological and social well-being are recognised as key developmental domains that should be evaluated using both positive indicators (e.g., social competence and prosocial behaviour) and negative indicators (e.g., emotional and behavioural difficulties) to provide a comprehensive understanding of children’s functioning [4]. Given the central role of socio-emotional development in child well-being, increasing attention has been directed towards preventive approaches that promote children’s socio-emotional development during the early years. Since these early developmental experiences occur primarily within the family, parents represent the most influential agents in promoting children’s socio-emotional well-being through everyday interactions and responsive caregiving [5]. Consequently, understanding how parents can be effectively supported in this role has become an important research priority, leading to the development of parent-focused interventions designed to strengthen parents’ capacities to promote their children’s socio-emotional well-being [6].

### 1.1. Socio-Emotional Well-Being in Early Childhood

Children’s well-being has become a major priority in contemporary public health, developmental psychology, and early childhood education, reflecting the growing recognition that healthy development extends beyond the absence of disease or developmental difficulties. Within this multidimensional framework, socio-emotional well-being has emerged as a fundamental dimension of healthy child development because it reflects children’s capacity to establish positive relationships, regulate emotions and behaviour, participate successfully in everyday social contexts, and engage meaningfully in learning experiences [7,8].

Early childhood represents a particularly sensitive developmental period for the promotion of socio-emotional well-being. From infancy through the preschool years, children undergo rapid neurological, cognitive, emotional and social development, during which the foundations of later psychological adjustment, learning and interpersonal functioning are established [9,10,11,12]. Because developmental processes during this period are highly responsive to children’s early experiences and environmental influences, the preschool years offer a unique opportunity to strengthen protective factors that support healthy developmental trajectories before emotional and behavioural difficulties become established [13]. Consequently, contemporary developmental and public health perspectives increasingly advocate preventive approaches that promote children’s socio-emotional well-being from the earliest years of life rather than focusing exclusively on the treatment of later difficulties [7,8].

Rather than referring solely to the absence of emotional or behavioural problems, socio-emotional well-being encompasses a broad range of interrelated competencies that enable children to function effectively within their social environments. These competencies include recognising, expressing, and regulating emotions, developing empathy, establishing and maintaining positive relationships, cooperating with others, demonstrating prosocial behaviour and responding adaptively to everyday social challenges [7,14]. As these competencies develop throughout early childhood, they collectively contribute to children’s psychosocial adjustment and social competence—two core indicators of healthy socio-emotional functioning that facilitate successful participation in family, school, and community settings [13].

A substantial body of evidence demonstrates that socio-emotional well-being during early childhood is associated with both immediate and long-term developmental outcomes. Children who display stronger socio-emotional competencies are more likely to experience positive school adjustment, higher academic achievement, healthier peer relationships and better psychological well-being throughout childhood and adolescence [9,15]. Conversely, deficits in socio-emotional functioning have been linked to behavioural difficulties and other adverse developmental outcomes [16]. Accordingly, promoting socio-emotional well-being during the preschool years is increasingly recognised as an essential investment in children’s lifelong health, educational success and overall quality of life [8,13].

### 1.2. Parents as Promoters of Children’s Socio-Emotional Well-Being

Children’s socio-emotional well-being develops within a network of interacting environmental contexts, among which the family represents the earliest and most influential setting. Ecological perspectives emphasize that development is shaped through continuous interactions between children and their immediate environments, with parents serving as the primary agents who influence children’s early emotional, social and behavioural experiences [5,17]. During the preschool years, everyday parent–child interactions provide the context in which children first learn how emotions are expressed, understood and regulated, while simultaneously acquiring the social skills required to build positive relationships and adapt successfully to their environments [18,19,20].

Parents’ influence on children’s social-emotional well-being extends beyond the provision of basic care and encompasses the quality of everyday interactions, emotional responsiveness and parenting behaviours. Attachment theory proposes that warm, responsive and emotionally available caregiving fosters secure attachment relationships, which provide children with a foundation for exploring their environment, regulating emotions and developing positive social relationships [21,22,23]. Similarly, ecological and social learning perspectives suggest that children acquire socio-emotional competencies through repeated interactions with significant adults, by observing parental behaviours, internalising emotional responses and practicing newly acquired skills within supportive family environments [17,24].

Parents promote children’s socio-emotional well-being through multiple interconnected processes. First, they serve as emotional models, demonstrating how emotions can be expressed and regulated in everyday situations [18,25]. Second, they actively support children’s emotional development by responding to children’s emotional expressions, discussing emotions and guiding children in understanding the causes and consequences of emotional experiences [26]. Supportive parental responses, characterised by warmth, encouragement and emotional coaching, have consistently been associated with greater emotional competence, more effective emotion regulation and higher levels of prosocial behaviour among preschool children [27,28]. In contrast, dismissive, punitive or emotionally unsupportive parenting practices have been linked to poorer emotion regulation, increased emotional distress and greater behavioural difficulties [29,30].

Beyond emotional development, parenting practices also play a central role in fostering children’s social competence and psychosocial adjustment. Positive parenting characterised by sensitivity, acceptance, and consistent support has been associated with higher levels of cooperation, empathy, peer acceptance, and adaptive social behaviour [31,32]. Conversely, rejecting or highly negative parenting has been associated with poorer social competence and less adaptive behavioural functioning [33]. Through everyday interactions, parents create opportunities for children to practise communication, cooperation, conflict resolution, and self-regulation, thereby facilitating their successful adjustment across home, school and community contexts [19,20].

Collectively, the available evidence indicates that parents represent one of the most influential and modifiable factors associated with children’s socio-emotional well-being during early childhood [5,26,32]. By creating emotionally supportive family environments and engaging in responsive parenting practices, parents promote children’s emotional competence, social competence and psychological adjustment, thereby supporting their overall well-being [18,19,25]. These findings provide a strong theoretical and empirical rationale for developing parent-focused interventions that strengthen parents’ capacity to support their children’s socio-emotional well-being through everyday family interactions [7,13].

### 1.3. Parent-Focused Interventions and Child Outcomes

Over the past three decades, increasing recognition of the pivotal role of parents in children’s socio-emotional development has led to the development of numerous parent-focused interventions designed to strengthen parenting practices and improve children’s developmental outcomes. These interventions are founded on the premise that empowering parents with knowledge, practical skills, and effective strategies enhances the quality of everyday parent–child interactions and, consequently, promotes children’s socio-emotional well-being [6,34,35].

Although parent-focused interventions vary considerably in their theoretical orientation, delivery format, duration, and cultural context, they share a common objective: strengthening parents’ capacity to support their children’s emotional, social and behavioural development [6,36]. Most programmes are grounded in well-established developmental theories, including Attachment Theory, Social Learning Theory and Bronfenbrenner’s Bioecological Theory [17,21,24], all of which emphasise the importance of responsive parenting, positive parent–child relationships, modelling and emotionally supportive family interactions [37,38,39]. Across programmes, common intervention components include parent education, modelling of positive parenting practices, role-playing, guided discussions, coaching, home-based activities, and structured parent–child interactions, all of which aim to strengthen parents’ confidence and competence in supporting their children’s socio-emotional development [6,37,40,41].

The effectiveness of parent-focused interventions has been consistently supported by empirical evidence. Systematic reviews and intervention studies indicate that participation in these programmes improves parenting practices, parental self-efficacy, emotional responsiveness, and the quality of parent–child relationships [6,35,36,42]. In parallel, positive effects have also been reported for children, including enhanced emotion regulation, emotional and social competence, prosocial behaviour, school readiness and psychosocial adjustment, together with reductions in emotional and behavioural difficulties [37,38,43,44]. Collectively, these findings suggest that strengthening parents’ everyday interactions with their children represents an effective pathway for promoting children’s socio-emotional well-being [6,35].

Established parent-focused interventions differ in their primary aims and modes of delivery. For example, programmes such as the Incredible Years and Triple-P place substantial emphasis on strengthening parenting skills and promoting positive parenting, while also addressing children’s behavioural, emotional, and social development [37,38]. Other interventions, such as Tuning in to Kids, have a more specific focus on emotion-focused parenting and children’s emotional competence [39]. *Hug and Change* builds on this broader evidence base while adopting an integrated focus on preschool children’s socio-emotional well-being. The programme combines parent-focused learning with immediate guided parent–child practice within each session and home-based activities, while addressing a broad range of socio-emotional competencies, including emotional understanding and regulation, empathy, communication, cooperation, conflict resolution, self-esteem, self-confidence and positive behaviour. Importantly, the programme was developed for implementation within Greek preschool settings, where empirically evaluated parent-focused interventions specifically addressing preschool children’s socio-emotional well-being remain limited. Thus, *Hug and Change* was developed to provide a structured, theory-informed and contextually relevant approach to supporting socio-emotional development during preschool years.

### 1.4. The Present Study

Against this background, the present study evaluated the effectiveness of *Hug and Change,* a structured parent-focused intervention programme developed for Greek families of preschool children. The programme was designed to support parents in promoting their children’s socio-emotional development through positive parent–child interactions, emotional communication and evidence-based parenting practices. Using a quasi-experimental pretest–posttest design, the study examined whether participation in the programme was associated with improvements in children’s psychosocial adjustment and social competence compared with a comparison group that received no intervention. By evaluating the *Hug and Change* programme, a parent-focused intervention developed specifically for the Greek context, this study aims to contribute to the growing international evidence on parenting interventions that promote children’s socio-emotional well-being and may inform the development of future family-based prevention initiatives. Specifically, the study addressed the following research questions:

RQ1. Does participation in the *Hug and Change* programme improve children’s psychosocial adjustment?

RQ2. Does participation in the *Hug and Change* programme improve children’s social competence?

Based on previous evidence demonstrating the positive effects of parent-focused interventions on children’s psychosocial adjustment and social competence, the following hypotheses were formulated:

**H1.**  *There will be differences in children’s emotional and behavioural difficulties and prosocial behaviour between the intervention and comparison groups following the intervention.*

**H2.**  *There will be differences in children’s social competence between the intervention and comparison groups following the intervention.*

## 2. Materials and Methods

### 2.1. Study Design

The present study employed a quasi-experimental pretest–posttest design with an intervention group and a comparison group to evaluate the effectiveness of the *Hug and Change* programme. Quasi-experimental designs of this type are widely applied in educational and social science research when interventions are delivered under real-world conditions and random allocation of participants is not feasible [45,46]. This design allowed the evaluation of intervention-related changes within an authentic preschool setting while retaining a comparison group against which programme effects could be compared.

### 2.2. Participants and Procedures

Participants were recruited using convenience sampling. To be eligible for participation, parents were required to (a) have at least one preschool-aged child enrolled in a participating preschool, (b) be able to attend the intervention sessions and complete the study assessments, and (c) not participate in another parenting intervention programme during the study period. Parents recruited from preschools in which the *Hug and Change* programme was delivered constituted the intervention group, whereas parents recruited from preschools located in a different area constituted the comparison group. The comparison group did not receive any parent-focused intervention programme during the study period.

A total of 123 parent–child dyads participated in the study. The intervention group comprised 61 parent–child dyads (n = 61), whereas the remaining participants formed the comparison group (n = 62). Parents in the intervention group were predominantly mothers (n = 55) with a mean age of 37.8 years (SD = 4.28); fathers (n = 6) had a mean age of 43.0 years (SD = 6.29). Comparable demographic characteristics were observed in the comparison group (see Table 1).

Children were 4 to 5 years old (intervention group: M_age_ = 4.95 years, SD_age_ = 0.60; comparison group: M_age_ = 5.14 years, SD_age_ = 0.52), corresponding to the preschool age range targeted by the programme (Table 2).

Data were collected at two time points. Baseline (T1) assessments were completed approximately 1–2 weeks before the start of the intervention sessions, and post-intervention (T2) assessments were completed 1–2 weeks after the programme ended. Parents in the comparison group completed the same questionnaire on an equivalent schedule. Questionnaires were distributed in closed envelopes together with a cover letter explaining the purpose of the study and were returned directly to the first investigator; parents who did not return the questionnaire within two weeks were contacted by telephone or e-mail through the preschool teachers as a reminder. All questionnaires distributed at T1 and T2 were completed and returned by both groups, and no participant attrition occurred between the two assessment points.

### 2.3. Measures

#### 2.3.1. Children’s Psychosocial Adjustment

Children’s psychosocial adjustment was assessed using the parent version of the Strength and Difficulties Questionnaire (SDQ) developed by Goodman [47]. The SDQ is a widely used screening instrument for assessing emotional and behavioural functioning in children and adolescents aged 3–16 years and has been extensively validated across different countries and populations [47,48,49,50]. The questionnaire consists of 25 items organised into five subscales: emotional symptoms, conduct problems, hyperactivity/inattention, peer problems and prosocial behaviour, with five items in each subscale. Responses are rated on a 3-point Likert scale (0 = Not true, 1 = Somewhat true, and 2 = Certainly true). For the needs of this study, two composite scores were examined: (a) behavioural difficulties score (derived from the four subscales of behavioural problems) and (b) a prosocial behaviour score. Higher scores on the behavioural difficulties indicate greater psychosocial difficulties, whereas higher scores on the prosocial behaviour subscale indicate more positive social behaviour. Consistent with the original scoring guidelines, negatively worded items were reverse-coded prior to computing composite scores; for the SDQ, items 7, 11, 14, 21, and 25 were reverse-scored and relabelled 7R, 11R, 14R, 21R, and 25R.

The SDQ demonstrated satisfactory psychometric properties in both clinical and community samples [48,49,51,52]. Previous studies have reported good internal consistency for the total difficulties score, with Cronbach’s alpha values ranging from 0.79 to 0.85 for parent reports [53,54]. The Greek version of the SDQ parent form was used in the present study [47].

#### 2.3.2. Children’s Social Competence

Children’s social competence was assessed using the Social Competence subscale of the parent-report Early School Behaviour Scale (ESBS) [55]. In the present study, only the social competence subscale was administered, as it specifically assesses children’s positive social skills, adaptive behaviours and their ability to interact effectively with others. This subscale was selected as it corresponds directly to the primary outcome of the present intervention. The subscale consists of 16 items rated on a 4-point Likert scale, ranging from 1 (almost never) to 4 (almost always). Higher scores indicate higher levels of children’s social competence. Previous studies have demonstrated satisfactory psychometric properties for the scale, with Cronbach’s alpha coefficients ranging from 0.79 to 0.89 for the parent version [52,53,54,56,57].

Table 3 presents the internal consistency of both SDQ and Social Competence subscales across time. For this study, McDonald’s omega coefficient was computed instead of Cronbach’s alpha coefficient, as it does not assume tau-equivalence among items and yields a less biased reliability estimate for Likert-type scales with unequal item loadings [58].

### 2.4. Intervention Programme Hug and Change

The *Hug and Change programme* [(in Greek, *Aγκαλιάζουμε και Aλλάζουμε*)] is a structured parent-focused programme developed for families of preschool children (aged 4–5 years old) to strengthen parents’ capacity to support their children’s socio-emotional development through everyday parent–child interactions. The programme focuses on preschool years, as this period is critical and sensitive for children’s socio-emotional development and well-being. During this time, children develop core capacities for emotion regulation, empathy, social competence, and prosocial behaviour and are strongly influenced by the quality of family relationships [59,60].

The design and evaluation of the programme were conducted as part of the principal investigator’s doctoral dissertation [61]. Following an extensive review of the literature on parent-focused interventions, the programme was developed to address the needs of Greek preschool families and educational settings [6]. The programme was theoretically based on Attachment Theory [21], which emphasises the importance of strengthening secure parent–child relationships; Bronfenbrenner’s Bioecological Theory [17], which highlights the influence of family interactions on child development; Bandura’s Social Cognitive Theory [24], which guided the use of modelling and observational learning and the Theory of Change [62], which provided the framework for the design, implementation and evaluation of the intervention. In addition, the programme content and learning objectives were organised according to the CASEL framework for Social and Emotional Learning [63] and the ORIM model, which informed the selection and sequencing of socio-emotional competencies and strategies for promoting daily parent–child interactions [64].

The programme consisted of 10 weekly face-to-face, group sessions of approximately 60 min, delivered to four parents’ groups of about 15 parents each. Sessions were conducted in the participating preschool settings and followed a specific structure. Each session consisted of two 30 min components: (1) a facilitator-led parent session addressing the weekly topic through discussion, modelling and role-play, while children took part in a supervised activity in a separate room led by a preschool teacher assistant; (2) a joint parent–child component in which newly introduced strategies were practiced through guided play and shared activities.

To provide more concrete illustration of how the programme components were implemented, the session focusing on emotional regulation included discussion with parents about supportive ways of responding to children’s intense emotions. Parents discussed strategies such as recognising and labelling emotions, accepting children’s emotional experiences, providing guidance, and modelling appropriate regulation strategies. Parents were introduced to practical emotion-regulation techniques, including the Anger Thermometer, breathing exercises and taking a short break. These strategies were also incorporate into guided parent–child activities and encouraged for use in everyday family situations.

To further support learning beyond the intervention sessions, parents received a structured handbook containing concise theoretical information, practical parenting strategies and home-based activities designed to reinforce positive parent–child interactions and facilitate the generalisation of acquired skills within the family context. Parents were encouraged to implement the suggested home activities between sessions and reflect on their experience during subsequent meetings, thereby promoting continuity of learning and skill application across the intervention period.

All sessions were delivered by the principal investigator, who also developed the programme, following a standardised intervention manual to promote consistency in programme delivery across groups. Participant attendance was recorded by the facilitator throughout the intervention period. The programme addressed ten core socio-emotional domains, including understanding emotions, emotional expression, emotional regulation, empathy, positive communication, conflict resolution, cooperation, self-esteem and self-confidence, positive behaviour and self-regulation.

### 2.5. Ethical Considerations

The study protocol was reviewed and approved by the Research Ethics and Quality Assurance Committee of the School of Social Sciences, International Hellenic University (protocol no. ΣΦ30/40/14-10-2024), in accordance with Article 70 of Greek Law 4485/2017. Prior to data collection, preschool directors, teachers, and parents were informed in writing about the purpose of the study, the data collection procedures, their rights, and the voluntary nature of participation. All participating parents provided written informed consent. Anonymity and confidentiality of personal data were ensured throughout the study, and participants were informed that they could withdraw at any time without any consequence.

### 2.6. Data Analysis Plan

Data were analysed using JAMOVI (version 2.3; The jamovi project, Sydney, Australia). The analysis proceeded in two stages—descriptive and inferential statistics—with the level of statistical significance set at *p* < 0.05 for all tests. Prior to the main analyses, the dataset was screened for duplicate participant codes, out-of-range values, and missing data, following standard data-cleaning procedures [58]. Descriptive statistics (frequencies, percentages, means, and standard deviations) were used to summarise participants’ demographic characteristics. Baseline equivalence between the intervention and comparison groups on demographic variables was examined using the independent-samples *t*-test for continuous variables (e.g., child and parent age) and the chi-square test for categorical variables (e.g., parent/child gender, educational level, employment status).

**Figure A1:**
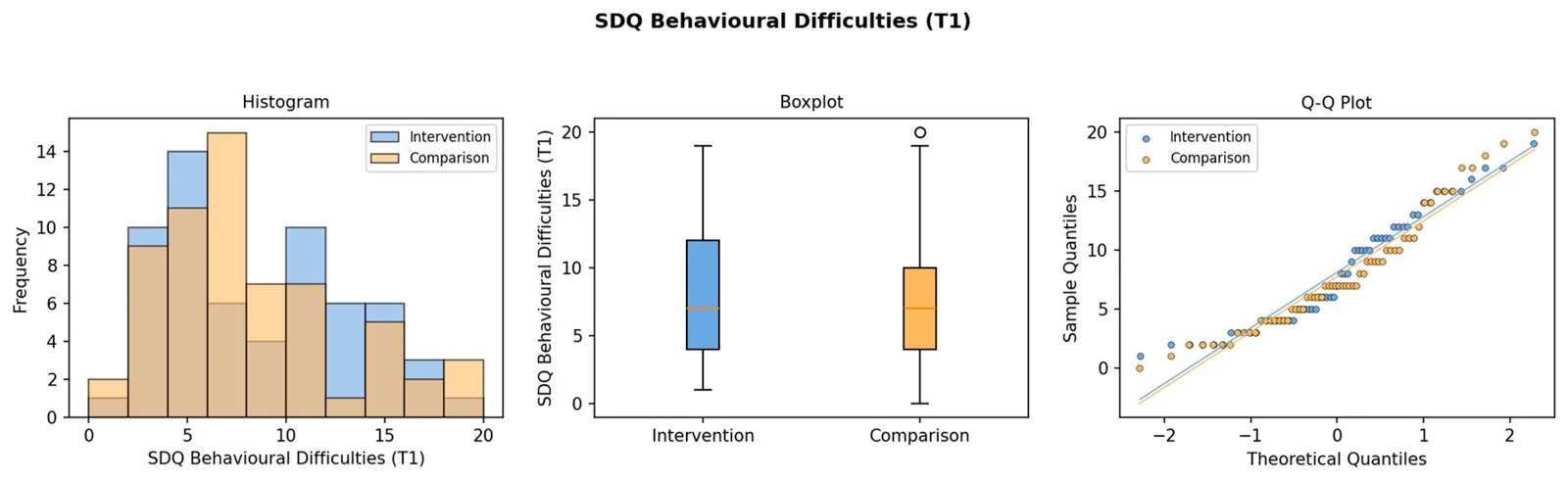
SDQ Behavioural Difficulties at pre-intervention (T1), by group. (**Left**) histogram; (**Centre**) boxplot; (**Right**) Q–Q plot of standardised residuals against theoretical quantiles.

**Figure A2:**
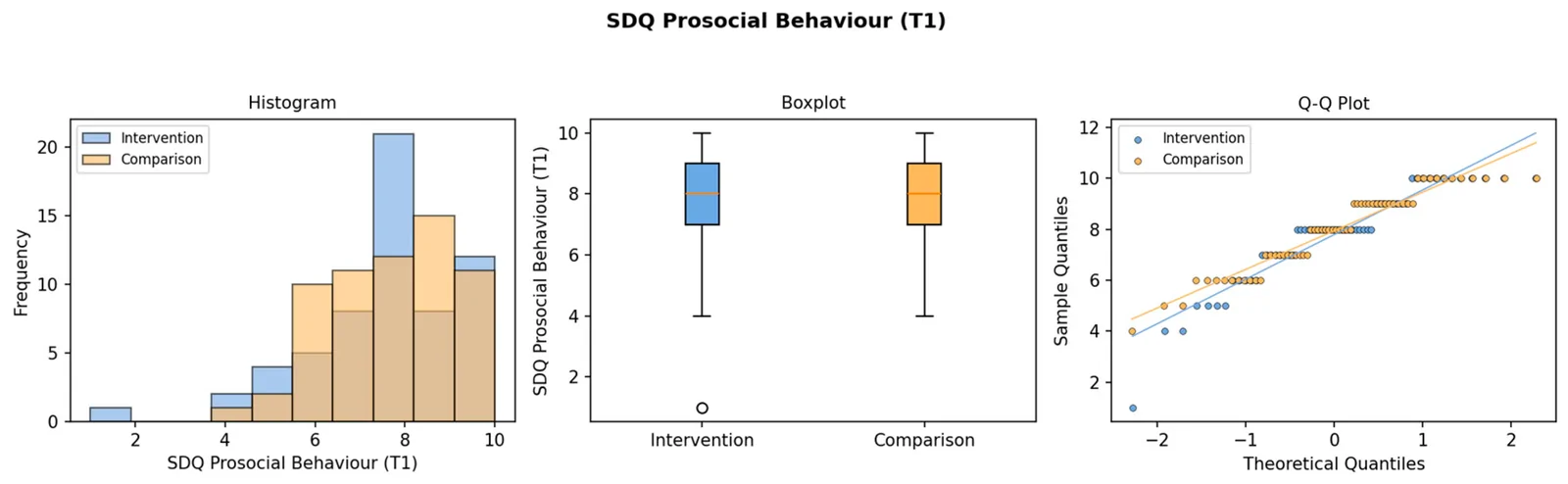
SDQ Prosocial Behaviour at pre-intervention (T1), by group. (**Left**) histogram; (**Centre**) boxplot; (**Right**) Q–Q plot of standardised residuals against theoretical quantiles.

**Figure A3:**
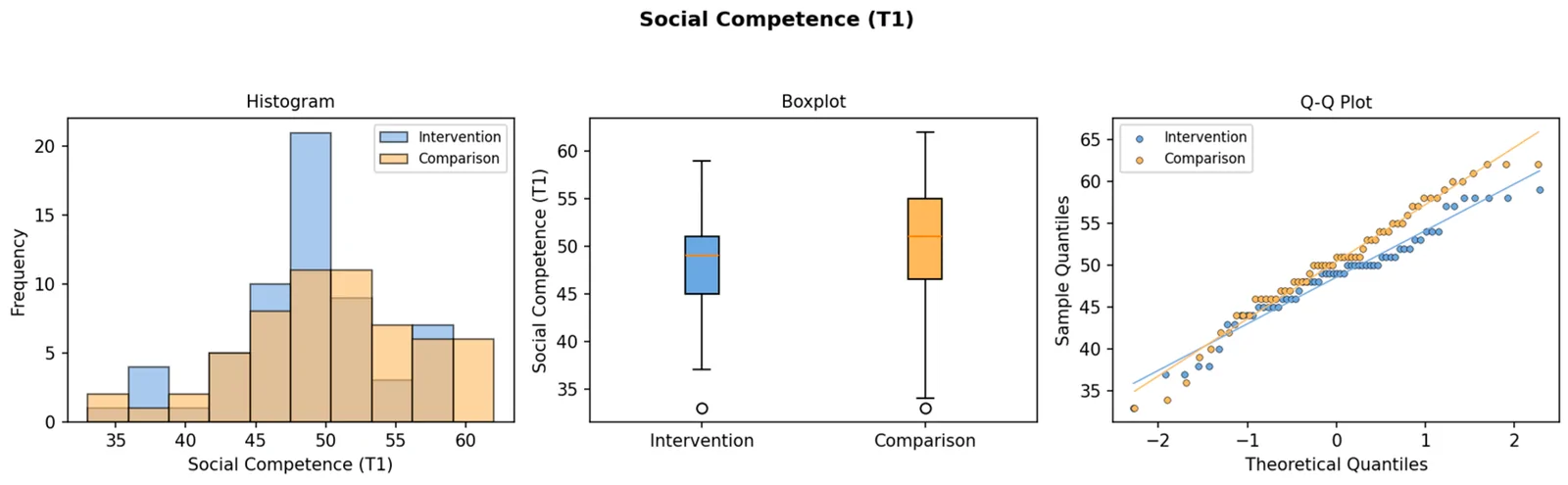
Social Competence at pre-intervention (T1), by group. (**Left**) histogram; (**Centre**) boxplot; (**Right**) Q–Q plot of standardised residuals against theoretical quantiles.

**Figure A4:**
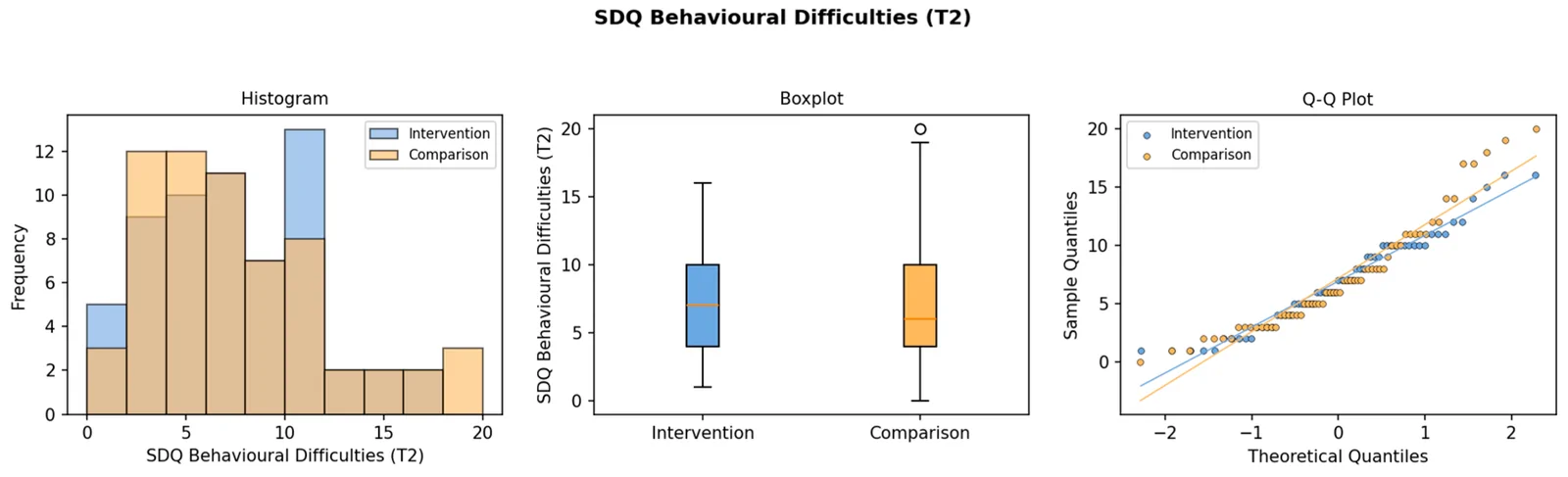
SDQ Behavioural Difficulties at post-intervention (T2), by group. (**Left**) histogram; (**Centre**) boxplot; (**Right**) Q–Q plot of standardised residuals against theoretical quantiles.

**Figure A5:**
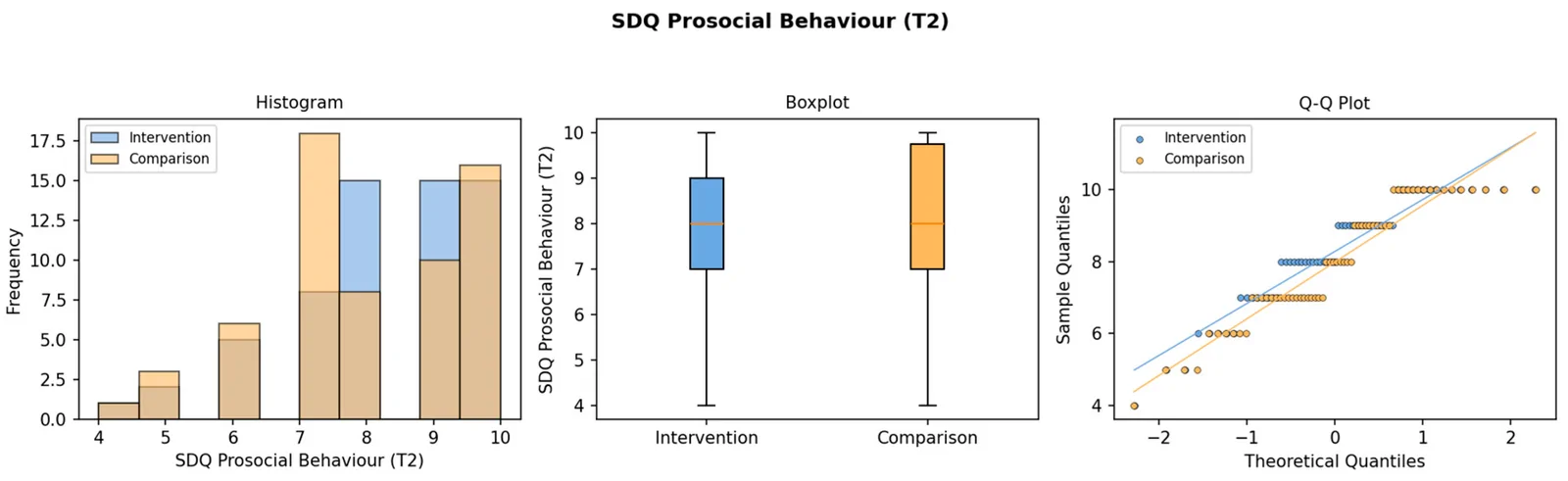
SDQ Prosocial Behaviour at post-intervention (T2), by group. (**Left**) histogram; (**Centre**) boxplot; (**Right**) Q–Q plot of standardised residuals against theoretical quantiles.

**Figure A6:**
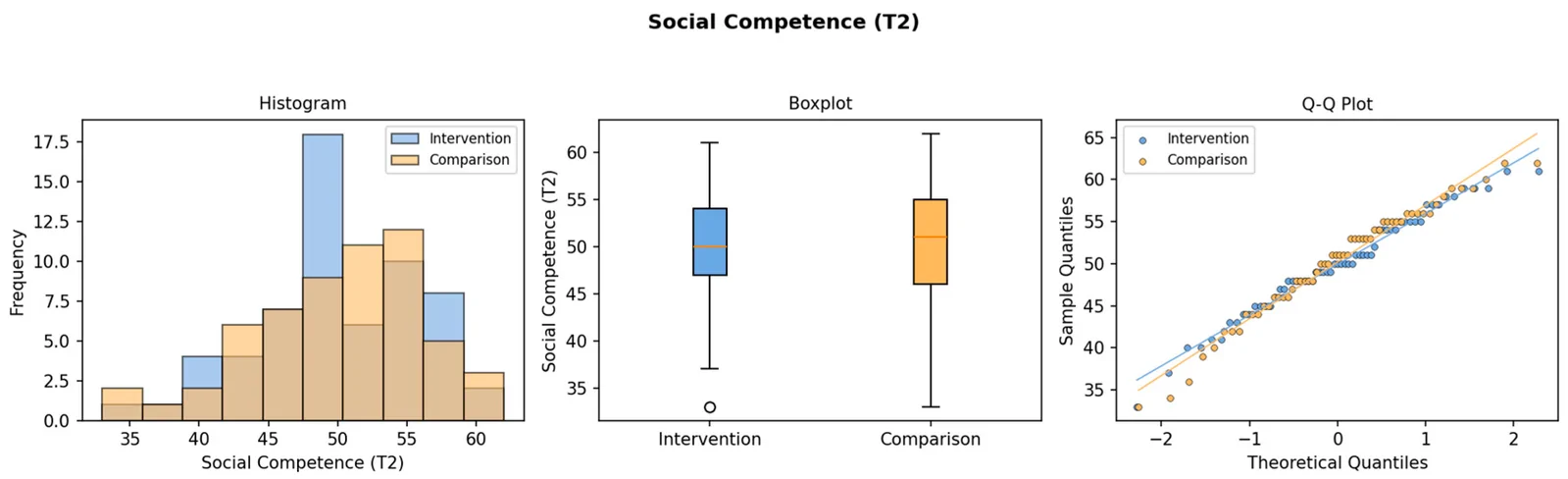
Social Competence at post-intervention (T2), by group. (**Left**) histogram; (**Centre**) boxplot; (**Right**) Q–Q plot of standardised residuals against theoretical quantiles.

The normality of the distribution of the study variables was examined using a variety of approaches. More specific, the Shapiro–Wilk test—considered particularly appropriate for small-to-moderate sample sizes—together with skewness and kurtosis indices, and through the visual inspection of histograms, boxplots, and Q–Q plots for each variable, separately for the intervention and comparison groups at both assessment points (see Appendix A Figure A1, Figure A2, Figure A3, Figure A4, Figure A5 and Figure A6). This combined approach was adopted to determine the suitability of parametric versus non-parametric procedures [58]. The majority of variables showed statistically significant departures from normality on the Shapiro–Wilk test and displayed visible asymmetry, skew, or outliers in the corresponding histograms, boxplots, and Q–Q plots, thus, non-parametric procedures were used throughout the analyses (Appendix A Table A1 and Figure A1, Figure A2, Figure A3, Figure A4, Figure A5 and Figure A6).

Given the two-wave (T1–T2) design of the study, change over time was operationalised using difference scores (T2–T1), analysed with the Wilcoxon signed-rank test (with-in-group) and the Mann–Whitney U test (between-group). For a two-wave design, this approach is equivalent to testing the group × time interaction estimated by a mixed-effects model or repeated-measures ANOVA, while avoiding distributional assumptions (e.g., normality of residuals) that the present data did not meet. Subsequently, a three-step analytic sequence was followed to evaluate change over time and between groups:

**Step 1—Computation of difference scores (T2–T1).** For each dependent variable, a new variable was computed representing the difference between the two assessment points, allowing direct estimation of the direction and magnitude of change for each participant. These difference scores served as the primary variables for the between-group comparisons.

**Step 2—Within-group comparisons (Wilcoxon signed-rank test).** To examine whether statistically significant change occurred within each group (intervention and comparison) from T1 to T2, the non-parametric Wilcoxon signed-rank test for paired samples was applied, as the non-parametric alternative to the paired-samples *t*-test when the assumption of normality is not met. Comparisons were conducted separately for each outcome (SDQ Behavioural Difficulties, SDQ Prosocial Behaviour, Social Competence), filtering the dataset by group.

**Step 3—Between-group comparison of difference scores (Mann–Whitney U test).** To determine whether the change observed in the intervention group differed significantly from that in the comparison group, the Mann–Whitney U test was applied to each difference variable, as the non-parametric alternative to the independent-samples *t*-test [65].

Non-parametric longitudinal alternatives (e.g., the Friedman test) were not used, as they target three or more repeated assessments rather than the two-wave design used here. The Wilcoxon–Mann–Whitney sequence therefore offered a transparent, assumption appropriate strategy for evaluating within- and between-group change, consistent with recommended practice for two-wave designs with non-normally distributed outcomes [58,65].

The significance level was set at *p* < 0.05 for all tests, and effect sizes were calculated for each comparison to estimate the magnitude of the observed differences.

## 3. Results

### 3.1. Initial Analyses

Demographic equivalence was examined using the independent-samples *t*-test for continuous variables and the chi-square test for categorical variables. No statistically significant differences were found between the two groups in parent age [*t*(120) = −1.29, *p* = 0.20], child age [*t*(121) = −1.91, *p* = 0.06], parent sex [*χ*^2^(1) = 1.61, *p* = 0.20], child sex [*χ*^2^(1) = 0.01, *p* = 0.93], mother’s educational level [*χ*^2^(4) = 1.55, *p* = 0.82], father’s educational level [*χ*^2^(5) = 5.13, *p* = 0.40], or family monthly income [*χ*^2^(9) = 13.9, *p* = 0.18]. These results indicate that the two groups were comparable on the basic socio-demographic characteristics at the beginning of the intervention.

Next, the differences between the groups regarding the baseline (T1) variables using the non-parametric Mann–Whitney U test were examined. The results of the analyses did not reveal statistically significant differences between the two groups on any of the variables (*p* > 0.05) (Table 4).

Table 5 presents means and standard deviations for the SDQ Behavioural Difficulties and Prosocial Behaviour scores, and for the Social Competence score, by group and assessment time.

### 3.2. Effects of the Intervention on SDQ Outcomes (RQ1)

To answer the first research question, changes in children’s behavioural difficulties and prosocial behaviour were examined separately within each group before being compared between groups. More specifically, a difference score (T2–T1) was first computed for each SDQ outcome. Within-group comparisons using the Wilcoxon signed-rank test showed that, in the intervention group, parents reported a statistically significant decrease in children’s Behavioural Difficulties from T1 to T2 (W = 171, *p* < 0.001, r = −0.670), and a statistically significant increase in children’s Prosocial Behaviour (W = 431, *p* < 0.001, r = 0.631). The comparison group, by contrast, showed no meaningful change in either behavioural difficulty (W = 252, *p* = 0.196, r = −0.246) or prosocial behaviour (W = 279, *p* = 0.786, r = 0.054) (Table 6).

Furthermore, between-group comparisons of the T2–T1 difference scores using the Mann–Whitney U test indicated that the decrease in Behavioural Difficulties was significantly greater in the intervention group than in the comparison group (U = 1311, *p* = 0.003). Similarly, the increase in Prosocial Behaviour was significantly greater in the intervention group than in the comparison group (U = 1516, *p* = 0.043) (Table 7).

### 3.3. Effects of the Intervention on Social Competence (RQ2)

Using the same three-step analytic sequence, the Wilcoxon signed-rank test showed a statistically significant increase in parent-reported Social Competence from T1 to T2 in the intervention group (W = 565, *p* = 0.005, r = 0.524). In the comparison group, the change in Social Competence from T1 to T2 was not statistically significant (W = 241, *p* = 0.869, r = 0.036) (Table 8).

Between-group comparison of the T2–T1 difference scores confirmed that the increase in Social Competence was significantly greater in the intervention group than in the comparison group (U = 1348, *p* = 0.029), with a moderate effect (r = 0.225) (Table 9).

## 4. Discussion

### 4.1. Effects on Children’s Psychosocial Adjustment

The present study demonstrated that participation in the *Hug and Change* programme was associated with significant improvements in preschool children’s psychosocial adjustment. Compared with the comparison group, children whose parents participated in the intervention reported fewer emotional and behavioural difficulties and higher levels of prosocial behaviour following programme completion. These findings support the first hypothesis and suggest that participation in the structure parent-focused intervention was associated with improvements in parent-reported psychosocial adjustment during the preschool years.

These findings are consistent with a growing body of evidence demonstrating that parent-focused interventions can improve children’s emotional and behavioural functioning. Recent systematic reviews and meta-analyses have concluded that parenting programmes contribute to reductions in emotional competence and behavioural adjustments, as well as reductions in internalizing and externalising difficulties [66,67,68]. Similar outcomes have been reported for well-established interventions including the Incredible Years programme [69,70], Triple-P [71], Tuning in to Kids [72,73] and Parent–child Interaction Therapy [74,75,76], all of which have demonstrated improvements in children’s psychosocial adjustment.

Although the overall pattern of findings is consistent with previous intervention research, the positive outcomes observed in the present study may also reflect several distinctive characteristics of the *Hug and Change* programme. Consistent with evidence-based recommendations for effective parent-focused interventions [6,34,35], the programme integrated parent education, guided parent reflection and immediate parent–child practice within the same session. This structure enabled parents not only to acquire new knowledge but also to apply newly learned strategies immediately through structured activities with their children under the guidance of the facilitator. Furthermore, the parent handbook and between-session home activities encouraged families to continue practising these strategies in everyday interactions, thereby facilitating the transfer of learning beyond the intervention setting. This integration of educational input, experiential training, guided practice and repeated application provided parents with repeated opportunities to apply the strategies introduced during the programme within structured parent–child activities and everyday family routines.

An important finding of this study was that the intervention produced a moderate effect on reducing children’s emotional and behavioural difficulties but only a small effect on increasing prosocial behaviour. This pattern is consistent with previous evaluations of parent-focused interventions, which have frequently reported stronger short-term effects for reducing behavioural difficulties than for enhancing positive social behaviours [76,77,78,79]. One possible explanation is that parents may implement behavioural management strategies relatively quickly, leading to immediate reductions in disruptive or challenging behaviours. In contrast, the development of prosocial behaviours, such as empathy, cooperation, helping and sharing, represents a more gradual developmental process that requires repeated opportunities for practice across different social contexts before stable behavioural changes become evident [78]. Consequently, the development of prosocial behaviour may require a longer period than that captured by the present short-term post-intervention assessment. Future studies incorporating follow-up assessments are needed to determine whether further changes emerge and weather the observed improvements are maintained over time.

Overall, the findings suggest that *Hug and Change* is a promising parent-focused intervention for promoting preschool children’s psychosocial adjustment. By providing parents with practical strategies and repeated opportunities to apply these strategies within meaningful parent–child interactions, the programme was associated with reductions in children’s emotional and behavioural difficulties and increases in positive social behaviour [79]. These findings reinforce the view that parents are key agents of change in early childhood interventions and highlight the importance of designing programmes that combine evidence-based parenting support with guided parent–child practice to promote children’s socio-emotional well-being.

### 4.2. Effects on Children’s Social Competence

The findings of the present study indicate that participation in the *Hug and Change* programme was associated with significant improvements in children’s social competence, supporting the second hypothesis. Children whose parents participated in the intervention were rated by parents as showing higher levels of social competence following programme completion compared with baseline and with children in the comparison group. These findings suggest that parent-focused interventions may support children’s ability to interact effectively with others and engage in positive social behaviours [80]. However, the between-group effect size for social competence was small (r = 0.225), indicating that the magnitude of the observed improvement was modest despite reaching statistical significance.

The present findings are consistent with a growing body of evidence demonstrating that parent-focused interventions can positively influence children’s social competence. Previous research has consistently shown that parenting programmes contribute to children’s social, emotional and behavioural development through changes in parenting behaviours and family interactions [35,68]. More recent meta-analytic evidence further supports the positive effects of parenting interventions on children’s social-emotional outcomes during early childhood [81]. In addition, social-emotional interventions for preschool children have been associated with improvements in social competence, with larger effects reported for incorporating a family components [82]. Similar improvements in children’s social competence have also been reported following participation in established parenting programmes, including Tuning in to Kids, the Incredible Years, the Social Emotional Prevention Program, and Triple-P, all of which have demonstrated positive effects on children’s interpersonal skills, emotional understanding and adaptive social functioning [71,72,73,83,84].

The observed improvements in children’s social competence may also be interpreted from an ecological perspective. According to Bronfenbrenner’s bioecological theory, children’s social development emerges through interactions with significant others within their everyday environments [17]. This interpretation is further supported by the Family Learning Ecosystem model [85], which views the family as the primary context in which social learning is continuously constructed through everyday experiences. From this perspective, supportive parent–child interactions may create opportunities for children to develop communication, cooperation, and interpersonal skills, thereby fostering the gradual development of social competence.

Overall, the findings suggest that the *Hug and Change* programme represents a promising parent-mediated intervention for promoting children’s social competence during the preschool years. Together with the improvements observed in psychosocial adjustment, these findings suggest that parent-focused interventions may support both behavioural adjustment and the development of adaptive social competencies, thereby contributing to children’s broader socio-emotional well-being.

### 4.3. Implications for Promoting Socio-Emotional Well-Being

The findings of the present study suggest that the *Hug and Change* programme promoted preschool children’s socio-emotional well-being by simultaneously reducing psychosocial difficulties and strengthening social competence. These findings support contemporary conceptualisations of socio-emotional well-being, which recognise well-being as a multidimensional construct encompassing not only the absence of emotional and behavioural difficulties but also the presence of positive social and emotional competencies that enable children to successfully engage with others and adapt to everyday challenges [86,87]. Taken together, the improvements observed across both outcome domains indicate that parent-focused interventions may contribute to children’s overall socio-emotional well-being rather than influencing isolated aspects of development [85].

The present findings further reinforce the central role of parents in promoting children’s socio-emotional well-being during early childhood. Previous research has shown that parent-focused interventions improve children’s developmental outcomes by enhancing parenting practices and the quality of everyday parent–child interactions [35,68]. These everyday family interactions can be viewed as meaningful learning opportunities through which children gradually develop social, emotional and behavioural competencies [85]. From this perspective, parent-focused interventions may provide an important approach to supporting children’s socio-emotional well-being.

The present study also contributes to the growing body of literature on culturally responsive parenting interventions. Although several evidence-based parenting programmes have demonstrated positive effects across different countries [6], relatively few interventions have been developed and empirically evaluated within the Greek early childhood context. These findings therefore suggest that parenting programmes grounded in established developmental theories and designed for local educational and cultural contexts may represent an effective strategy for promoting socio-emotional well-being during early childhood.

### 4.4. Practical Implications for Parents and Early Childhood Education

The findings of the present study have several practical implications for parents, educators, and policymakers. First, the findings highlight the importance of empowering parents as active partners in promoting children’s socio-emotional well-being rather than viewing them as recipients of parenting information. Providing parents with practical strategies that can be integrated into everyday family routines may represent an effective and sustainable approach to supporting young children’s emotional and social development.

Moreover, implementing programmes such as *Hug and Change* programme within early childhood education settings may strengthen family-school partnerships while increasing families’ access to evidence-based parenting support. Integrating similar programmes into preschool practice could complement existing socio-emotional learning initiatives and provide more consistent support for children’s well-being across family and school environments.

### 4.5. Strengths and Limitations

The present study has several strengths. It evaluated a theory-informed, culturally designed parent-focused intervention developed specifically for Greek preschool families using a quasi-experimental design with a comparison group and validated assessment instruments.

Several limitations should also be acknowledged. First, the use of convenience sampling and the absence of random allocation may have introduced selection bias. Although no significant differences were identified between the groups in the measured baseline characteristics, this does not establish group equivalence, as unmeasured characteristics may have differed between the groups and potentially influenced the observed outcomes. In addition, participants were recruited from two different locations, and contextual differences between these settings cannot be excluded as potential confounding factors. These limitations restrict causal interpretation of the findings and should be considered when evaluating the effectiveness of the programme.

Moreover, all outcomes were assessed solely using parent-report measures. As parents in the intervention group were aware of their participation in the programme and its aims, their post-intervention ratings may have been influenced by social desirability, expectancy effects, or reporting bias. Future studies should incorporate multiple informants, such as teachers, as well as independent observational measures to provide a more comprehensive assessment of children’s outcomes.

Furthermore, the post-intervention assessment was conducted approximately 1–2 weeks after programme completion; therefore, the findings reflect short-term post-intervention changes. The absence of longer-term follow-up assessments prevents conclusions regarding whether these changes were maintained over time. Relatedly, the two-wave design constrained the analytic approach to pre–post comparisons; studies with additional assessment points could instead apply linear mixed-effects models, which offer greater flexibility for modelling change over time.

In addition, parenting practices and the quality of parent–child interactions were not directly assessed; therefore, the mechanisms underlying the observed changes in child outcomes could not be examined.

Finally, although participant attendance was recorded and a standardised intervention manual was used to promote consistency in programme delivery, no formal fidelity assessment was conducted to systematically evaluate facilitator adherence, participation in programme components, or completion of home activities. This should be considered when interpreting the findings.

### 4.6. Future Research

Future research should examine whether the positive effects of the *Hug and Change* programme are maintained over time through longitudinal follow-up assessments. Evaluating the sustainability of intervention outcomes would provide valuable evidence regarding the long-term effectiveness of parent-focused interventions and the durability of improvements in children’s socio-emotional well-being. Replication studies involving larger and more diverse samples from different geographical regions and cultural contexts are also needed to strengthen the generalisability of the present findings.

In addition, the development and validation of an intervention fidelity measure would represent an important contribution to the field. Such a measure could assess the extent to which parents consistently implement the programme’s strategies within everyday family routines following programme completion. Monitoring implementation fidelity in the home environment would provide valuable insights into the transfer of newly acquired parenting practices into daily life and help identify the factors that support or hinder the long-term effectiveness and sustainability of parent-focused interventions. Future studies should also incorporate multiple sources of assessment, such as teacher reports or direct behavioural observations, to provide a more comprehensive evaluation of children’s socio-emotional development.

## 5. Conclusions

The present study provides preliminary evidence supporting the potential of the *Hug and Change* programme as a parent-focused intervention for promoting preschool children’s socio-emotional well-being. The findings indicate modest short-term improvements in children’s psychosocial adjustment and social competence, with between-group effects ranging from small to moderate. Parents who participated in the programme reported positive changes in their children’s psychosocial adjustment and social competence, suggesting that strengthening parenting practices and everyday parent–child interactions may contribute to positive socio-emotional developmental outcomes during early childhood.

These findings support the growing recognition that parents play a central role in fostering children’s socio-emotional well-being and highlight the value of preventive, theory-informed parent-focused interventions delivered within early childhood education settings. By combining evidence-based parenting strategies with opportunities for guided parent–child practice, the *Hug and Change* programme offers a theory-informed approach that is both feasible and applicable within the Greek preschool context.

Supporting parents during early childhood represents a promising strategy for fostering children’s socio-emotional well-being and may contribute to healthier developmental paths across childhood.

## Figures and Tables

**Table 1:** Demographic characteristics of parents in the intervention and comparison groups.

Characteristic	Intervention Group (n = 61)	Comparison Group (n = 62)
Parent sex		
Mother, n (%)	55 (90.2)	50 (80.6)
Father, n (%)	6 (9.8)	11 (17.7)
Mother age (years), M (SD)	37.8 (4.28)	38.0 (4.81)
Father age (years), M (SD)	43.0 (6.29)	46.5 (6.06)
Parent age range (years)	28–50	28–60
Number of children in family		
1, n (%)	24 (39.3)	22 (35.5)
2, n (%)	31 (50.8)	33 (53.2)
3, n (%)	6 (9.8)	7 (11.3)
Mother’s educational level		
Upper secondary, n (%)	4 (6.6)	6 (9.7)
Vocational/technical, n (%)	16 (26.2)	14 (22.6)
University, n (%)	30 (49.2)	29 (46.8)
Postgraduate, n (%)	11 (18.0)	11 (17.7)
Father’s educational level		
Lower secondary, n (%)	0 (0.0)	3 (4.8)
Upper secondary, n (%)	10 (16.4)	5 (8.1)
Vocational/technical, n (%)	15 (24.6)	13 (21.0)
University, n (%)	30 (49.2)	30 (48.4)
Postgraduate, n (%)	5 (8.2)	5 (8.1)
Doctoral, n (%)	1 (1.6)	3 (4.8)
Monthly household income (EUR)		
401–800, n (%)	2 (3.3)	3 (4.8)
801–1200, n (%)	4 (6.6)	5 (8.1)
1201–1600, n (%)	13 (21.3)	11 (17.7)
1601–2000, n (%)	11 (18.0)	14 (22.6)
2001–2400, n (%)	17 (27.9)	22 (35.5)
2401–2800, n (%)	8 (13.1)	1 (1.6)
2801–3200, n (%)	0 (0.0)	3 (4.8)
3201–3600, n (%)	3 (4.9)	1 (1.6)
3601–4000, n (%)	1 (1.6)	0 (0.0)
>4000, n (%)	0 (0.0)	2 (3.2)

Note. M = mean; SD = standard deviation.

**Table 2:** Demographic characteristics of children in the intervention and comparison groups.

Characteristic	Intervention Group (n = 61)	Comparison Group (n = 62)
Sex		
Male, n (%)	31 (50.8)	32 (51.6)
Female, n (%)	30 (49.2)	30 (48.4)
Age (years), M (SD)	4.95 (0.60)	5.14 (0.52)
Age range (years)	3.04–6.03	4.18–6.13
Birth order		
Only child, n (%)	25 (41.0)	22 (35.5)
First child, n (%)	18 (29.5)	13 (21.0)
Middle child, n (%)	4 (6.6)	6 (9.7)
Last child, n (%)	9 (14.8)	21 (33.9)
Other, n (%)	5 (8.2)	0 (0.0)
Nationality		
Greek, n (%)	61 (100.0)	61 (98.4)
Other, n (%)	0 (0.0)	1 (1.6)

Note. M = mean; SD = standard deviation.

**Table 3:** Internal consistency of the study’s measures across time.

Measure	T1	T2
SDQ—Behavioural Difficulties (parent report)	ω = 0.78	ω = 0.81
SDQ—Prosocial Behaviour (parent report)	ω = 0.68	ω = 0.70
Social Competence (parent report)	ω = 0.83	ω = 0.85

Note. T1 = pre-intervention assessment (baseline); T2 = post-intervention assessment. ω = Omega coefficient.

**Table 4:** Baseline (T1) comparison of outcome variables between the intervention and comparison groups.

Variable	U	*p*
SDQ—Behavioural Difficulties	1818	0.713
SDQ—Prosocial Behaviour	1853	0.847
Social Competence	1456	0.071

**Table 5:** Descriptive statistics for SDQ and Social Competence scores by group and time.

Variable	Group	T1		T2	
		M	SD	M	SD
SDQ—Behavioural Difficulties	Intervention (n = 61)	8.10	4.74	6.15	2.90
	Comparison (n = 62)	7.76	4.75	7.18	4.67
SDQ—Prosocial Behaviour	Intervention (n = 61)	7.79	1.81	8.38	1.33
	Comparison (n = 62)	7.94	1.54	7.98	1.61
Social Competence	Intervention (n = 61)	48.57	5.54	49.90	5.96
	Comparison (n = 62)	50.12	6.0	50.20	6.73

Note. M = mean; SD = standard deviation. T1 = pre-intervention assessment (baseline); T2 = post-intervention assessment.

**Table 6:** Within-group (T1–T2) comparisons for SDQ outcomes (Wilcoxon signed-rank test).

Variable	Group	W	*p*	r
Behavioural Difficulties	Intervention	171	<0.001	−0.670
Behavioural Difficulties	Comparison	252	0.196	−0.246
Prosocial Behaviour	Intervention	431	<0.001	0.631
Prosocial Behaviour	Comparison	279	0.786	0.054

Note. T1 = pre-intervention assessment (baseline); T2 = post-intervention assessment. Values are based on parent-report SDQ data. r = effect size interpreted as small ≈ 0.10, moderate ≈ 0.30, large ≈ 0.50 (Field, 2017 [58]). Negative r values for Behavioural Difficulties indicate a decrease in scores from T1 to T2; positive r values for Prosocial Behaviour indicate an increase.

**Table 7:** Between-group comparison of T2–T1 difference scores for SDQ outcomes (Mann–Whitney U test).

Variable	Group	M Diff	SD Diff	U	*p*	r
Behavioural Difficulties	Intervention	−1.95	3.34	1311	0.003	0.307
Behavioural Difficulties	Comparison	−0.58	2.60			
Prosocial Behaviour	Intervention	0.59	1.36	1516	0.043	0.198
Prosocial Behaviour	Comparison	0.06	1.13			

Note. M diff and SD diff = mean and standard deviation of the T2–T1 difference score for each group. U and *p*/r are reported once per variable, reflecting the single between-group comparison of the two groups’ difference scores. r = effect size, interpreted as small ≈ 0.10, moderate ≈ 0.30, large ≈ 0.50 (Field, 2017 [58]).

**Table 8:** Within-group (T1–T2) comparison for social competence (Wilcoxon signed-rank test).

Variable	Group	W	*p*	r
Social Competence	Intervention	565	0.005	0.524
Social Competence	Comparison	241	0.869	0.036

Note. T1 = pre-intervention assessment (baseline); T2 = post-intervention assessment.

**Table 9:** Between-group comparison of T2−T1 difference scores for social competence (Mann–Whitney U test).

Variable	Group	M Diff	SD Diff	U	*p*	r
Social Competence	Intervention	1.34	3.89	1348	0.029	0.225
Social Competence	Comparison	0.07	3.39			

Note. M diff and SD diff = mean and standard deviation of the T2–T1 difference score for each group. U and *p*/r are reported once per variable, reflecting the single between-group comparison of the two groups’ difference scores.

## Data Availability

The data presented in this study are available on request from the corresponding author due to ethical and privacy restrictions. The dataset contains participant-level data reported by parents for young children and is therefore not publicly available.

## Abbreviations

The following abbreviations are used in this manuscript:

ESBS	Early School Behaviour Scale
CASEL	Collaborative for Academic, Social, and Emotional Learning
SDQ	Strengths and Difficulties Questionnaire

## Appendix A

Table A1 presents skewness, kurtosis, and Shapiro–Wilk test results for the parent-report SDQ and Social Competence scores at pre-intervention (T1) and post-intervention (T2), separately for the intervention and comparison groups, supporting the decision to use non-parametric statistical procedures for all study analyses.

**Table A1:** Tests of normality for the parent-report SDQ and Social Competence scores at pre- (T1) and post-intervention (T2), by group (Shapiro–Wilk test).

Variable	Group	n	Skewness	SE	Kurtosis	SE	W	*p*
SDQ Behavioural Difficulties (T1)	Intervention	61	0.423	0.306	−0.966	0.604	0.935	0.003
SDQ Behavioural Difficulties (T1)	Comparison	62	0.808	0.304	0.076	0.599	0.935	0.003
SDQ Prosocial Behaviour (T1)	Intervention	61	−1.135	0.306	2.132	0.604	0.888	<0.001
SDQ Prosocial Behaviour (T1)	Comparison	62	−0.366	0.304	−0.690	0.599	0.925	<0.001
Social Competence (T1)	Intervention	61	−0.434	0.306	0.507	0.604	0.961	0.051
Social Competence (T1)	Comparison	62	−0.420	0.311	0.179	0.613	0.972	0.192
SDQ Behavioural Difficulties (T2)	Intervention	61	0.335	0.306	−0.479	0.604	0.958	0.034
SDQ Behavioural Difficulties (T2)	Comparison	62	0.982	0.304	0.551	0.599	0.920	<0.001
SDQ Prosocial Behaviour (T2)	Intervention	61	−0.752	0.306	0.098	0.604	0.900	<0.001
SDQ Prosocial Behaviour (T2)	Comparison	62	−0.263	0.304	−0.825	0.599	0.904	<0.001
Social Competence (T2)	Intervention	61	−0.324	0.306	0.102	0.604	0.979	0.392
Social Competence (T2)	Comparison	62	−0.544	0.314	0.024	0.618	0.970	0.161

Note. SE = standard error. W = Shapiro–Wilk test statistic. Skewness and kurtosis values are unstandardised (raw) statistics computed separately for the intervention (Group 1) and comparison (Group 2) groups, based on parent-report data, using the analysis software (JAMOVI, version 2.3). Most variable-by-group combinations showed statistically significant departures from normality (*p* < 0.05), supporting the use of non-parametric statistical procedures for all study analyses.
